# Importance of Ara h 6 in peanut allergy diagnosis

**DOI:** 10.1186/2045-7022-4-S2-O16

**Published:** 2014-03-17

**Authors:** Maria Pedrosa, Maria-Teresa Boyano-Martínez, Maria-Carmen García-Ara, Teresa Caballero, Santiago Quirce

**Affiliations:** 1 Hospital La Paz Institute for Health Research (IdiPAZ), Department of Allergy, Madrid, Spain

## Background

Seed storage proteins in peanut available for diagnostic purposes have included Ara h 1, Ara h 2, Ara h 3 and Ara h 8. Nevertheless, recent MA-ISAC 112 has incorporated Ara h 6 and Ara h 9 which may lead to an improved diagnosis.

## Methods

Ninety-one children have been included in the study. Data regarding clinical tolerance to peanut (PNT) have been assessed. Skin prick testing with PNT extract, LTP, profilin, as well as sIgE by means of ImmunoCAP and MA ISAC has been performed. ROC curves were constructed for the different diagnostic methods.

## Results

Twenty-two children (median age: 6.63 y.o.; IQR: 5.22-10.12) were classified as allergic in the basis of at least two convincing clinical reactions upon ingestion of PNT in the last two years. Sixty-nine children (median age: 8.01 y.o.; IQR: 6.01-11.27) were classified as tolerant on the basis of regular consumption of PNT. Positive SPT with commercial PNT extract was found in 86% of the allergic vs. 12% in the tolerant group (p=.000), sIgE for PNT extract was positive (>0.35 kU/L) in 100% of allergic (median: 10.40, IQR: 4.86-70.73) vs. 57% of tolerants (median: 1.69, IQR: 0.85-5.59) (p=.000). In MA-ISAC Ara h 6 was the allergen most frequently recognized (77%) (median: 8,50, IQR:4.29-33.70), followed by Ara h 2 (59%) (median: 17,30, IQR: 0.57-37.41). ROC curves showed the best diagnostic performance for Ara h 6 (AUC=0.852) followed by Ara h 2 (AUC=0.792). A cut-off point of Ara h 6 for maximal sensitivity (Se) and specificity (Sp) was set at 1.245 ISU (Se=0,727 and Sp= 0.971).

## Conclusions

Although Ara h 2 has been regarded as the marker of clinical reactivity in children allergic to peanut, special attention has to be paid to Ara h 6, which has been shown to have better diagnostic performance.

